# Effectiveness of a MF-59™-adjuvanted pandemic influenza vaccine to prevent 2009 A/H1N1 influenza-related hospitalisation; a matched case-control study

**DOI:** 10.1186/1471-2334-11-196

**Published:** 2011-07-18

**Authors:** Anneke Steens, Eleonora G Wijnans, Jeanne P Dieleman, Miriam CJM Sturkenboom, Marianne AB van der Sande, Wim van der Hoek

**Affiliations:** 1 Epidemiology and Surveillance Unit, Centre for Infectious Disease Control, National institute for Public Health and the Environment, Bilthoven, the Netherlands; 2 Department of Medical Informatics and Epidemiology, Erasmus University Medical Centre, Rotterdam, the Netherlands; 3 Medicines Evaluation Board, The Hague, the Netherlands; 4 Julius Centre for Health Research and Primary Care, University Medical Centre Utrecht, Utrecht, the Netherlands

## Abstract

**Background:**

During the 2009 influenza A/H1N1 pandemic, adjuvanted influenza vaccines were used for the first time on a large scale. Results on the effectiveness of the vaccines in preventing 2009 influenza A/H1N1-related hospitalisation are scanty and varying.

**Methods:**

We conducted a matched case-control study in individuals with an indication for vaccination due to underlying medical conditions and/or age ≥ 60 years in the Netherlands. Cases were patients hospitalised with laboratory-confirmed 2009 A/H1N1 influenza infection between November 16, 2009 and January 15, 2010. Controls were matched to cases on age, sex and type of underlying medical condition(s) and drawn from an extensive general practitioner network. Conditional logistic regression was used to estimate the vaccine effectiveness (VE = 1 - OR). Different sensitivity analyses were used to assess confounding by severity of underlying medical condition(s) and the effect of different assumptions for missing dates of vaccination.

**Results:**

149 cases and 28,238 matched controls were included. It was estimated that 22% of the cases and 28% of the controls received vaccination more than 7 days before the date of onset of symptoms in cases. A significant number of breakthrough infections were observed. The VE was estimated at 19% (95%CI -28-49). After restricting the analysis to cases with controls suffering from severe underlying medical conditions, the VE was 49% (95%CI 16-69).

**Conclusions:**

The number of breakthrough infections, resulting in modest VE estimates, suggests that the MF-59™ adjuvanted vaccine may have had only a limited impact on preventing 2009 influenza A/H1N1-related hospitalisation in this setting. As the main aim of influenza vaccination programmes is to reduce severe influenza-related morbidity and mortality from influenza in persons at high risk of complications, a more effective vaccine, or additional preventive measures, are needed.

## Background

Vaccination is the mainstay of preventing and mitigating the impact of influenza in spite of moderate vaccine effectiveness (VE). Lowering the burden of severe disease is one of the main aims of a vaccination program. As influenza can be a precipitating factor for the exacerbation of underlying medical conditions, an influenza vaccination strategy often targets specific risk groups. Unfortunately, VE is generally lower in individuals with a compromised immune system, such as the elderly [1].

Antigen supplies during a pandemic are expected to be limited. For that reason, in 2005 the WHO recommended adjuvanted vaccines in just such scenario [2]. Inclusion of an adjuvant enhances immunogenicity of a vaccine [3], thereby reducing the amount of antigen required for equivalent immune responses. During the recent 2009 influenza A/H1N1 (pH1N1) pandemic, adjuvanted influenza vaccines were used for the first time on a large scale in Europe. Five vaccines were authorised by the European Medicines Agency (EMA) for use in the European Union [4]. Clinical trials reported high immunogenicity of the adjuvanted vaccines [5-10]; post-marketing studies [11-16] showed an effectiveness on preventing confirmed pH1N1 that was similar to that of seasonal influenza vaccines in well matched years [11,17]. So far, only limited and varying results have been reported on the effectiveness of the adjuvanted vaccines in preventing severe disease of pH1N1 that required hospitalisation [13,14,18].

VE estimates with severe outcomes are important for guiding decisions on recommendations for complementary or alternative public health measures, and for communication to and preparedness of health care and the society. Studies estimating VE in specific risk groups are important, especially where new vaccines are used, as those groups are generally not included in clinical trials. Until now, none of the published studies on the effectiveness of the vaccines in preventing pH1N1-related hospitalisation [13,14,18] focussed on a MF-59™-adjuvanted vaccine. We investigated the effectiveness of a MF-59™-adjuvanted vaccine [19] in preventing pH1N1-related hospitalisation in individuals with an indication for vaccination due to underlying medical conditions and/or age ≥ 60 years in the Netherlands. Such a study was enabled by the mandatory notification of pH1N1 cases requiring hospitalisation. We combined the notification data with data of an extensive general practitioner (GP) network in the Netherlands (Integrated Primary Care Information (IPCI) database [20,21]) employing a matched case-control design.

## Methods

### Study design and setting

We conducted a matched case-control study. As hospitalisation for pH1N1 infection was notifiable, case-data were obtained through the routine infectious diseases surveillance system [22,23]. In the Netherlands, all patients hospitalised for suspected for pH1N1 infection were swabbed and tested. The instructions were to perform a nose and a throat swab combined in one transport medium. Laboratory confirmation was done by real-time PCR for influenza virus type A and type A(H1N1) [24]. Following laboratory confirmation, the attending physician and the laboratory had the legal requirement to contact the Municipal Health Service, who notified the case by entering the reported data into the national password-secured web-based routine surveillance database. Reported data included information on underlying medical conditions in broad categories (pulmonary disease, cardiac disease, diabetes mellitus, chronic kidney failure, cancer and immunocompromised condition) and self-reported vaccination status for the seasonal and pandemic vaccines. Missing data were retrieved through the hospital physicians; before discharge the hospital physician would ask the patient directly, while after discharge, GPs were contacted in case the GP of the patient was known. Control-data were available anonymously in the IPCI database. Details about the database have been reported elsewhere [20]. In short, the IPCI database is a longitudinal GP research database and contains electronic patient records of about 500 GPs from all over the Netherlands. This includes prescription data and specialists' letters. Currently there are over 750,000 active patients, representing approximately 5% of the Dutch population. As in the Netherlands, nearly all people are registered with a GP, the patient population is representative of the Dutch population regarding sex and age, except for a slight under representation of the elderly population that is under care of medical practitioners in nursing homes.

The IPCI database complies with European Union guidelines on the use of medical data for medical research. The Scientific and Ethical Advisory Board of the IPCI project approved the study. Informed consent was not required.

### Vaccination programme

In the Netherlands, different groups were eligible for a pandemic influenza vaccination in 2009: those with specified underlying medical conditions (pulmonary disease, cardiac disease, diabetes mellitus, chronic kidney failure, cancer, immunocomprised condition), pregnant women in their second and third trimester, institutionalised individuals, individuals aged ≥ 60 years, children aged 6 months to 4 years, health care workers with potential for direct patient contact, family members and caretakers of individuals with high risk for severe disease or death, and household members of children younger than 6 months.

The MF-59™ adjuvanted vaccine [19] was provided to individuals with underlying medical conditions and/or aged ≥ 60 years through the GP. Vaccination started in week 45 (November 2, 2009), though the majority of the GPs (99% of our control sample) provided vaccination from week 46 (November 9, 2009) onwards. All persons were offered two doses, two weeks apart. The 2009 influenza epidemic in the Netherlands started in week 41, peaked in week 46 with 190 cases per 100,000 inhabitants and ended in week 50 [25].

### Study population

**Figure 1:**
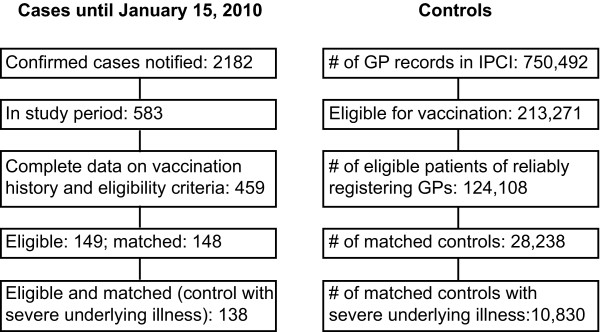
**Flow diagram of cases and controls**. Cases are derived from the routine infectious diseases surveillance system, controls originated from the IPCI GP database.

Cases were patients who had been hospitalised because of a laboratory confirmed pH1N1 infection. Only cases with day of symptom-onset between November 16, 2009 and January 15, 2010 and with information on vaccination history were included (see Figure 1). Pregnant women were not included in the analysis. The date of symptom onset of cases was used as the index date.

Patients from the IPCI database were eligible as controls when they had at least 1 year of valid database history available, when they were eligible for vaccination by the GP due to underlying medical conditions and/or advanced age, and when they were registered with a GP that had consistent and complete registration of vaccinations (58% of all GPs; see Figure 1). Underlying medical conditions were extracted from the IPCI database using International Classification of Primary Care (ICPC) codes as well as free text terms and were aggregated into categories to make the level of information of the cases and controls similar. Information on vaccination status and date of vaccination were extracted using algorithms based on ICPC code and open text fields including brands and batch-code. Consistent registration of vaccinations was defined as a coverage of the seasonal influenza vaccination, 1^st ^dose of pandemic influenza vaccine and 2^nd ^dose of pandemic vaccine of ≥ 50% in the GP practice population aged ≥ 60 years. This cut off was based on the national vaccination coverage estimate of the population aged ≥ 60 years in 2009 (respectively 76%, 77% and 69% [26]). From the selected eligible population, we sampled all possible controls matching a case on age (+/- 12 months), sex, underlying medical conditions (all conditions at aggregated level) and calendar date (i.e. were alive and present in the database for at least 1 year at the index date). One case did not have a matched control. Women with known confirmed pregnancy (ICPC codes; n = 43) and individuals that had been hospitalised with confirmed or suspected pH1N1 infection before the index date (open text fields; n = 17) were excluded.

### Exposure definition

**Figure 2:**
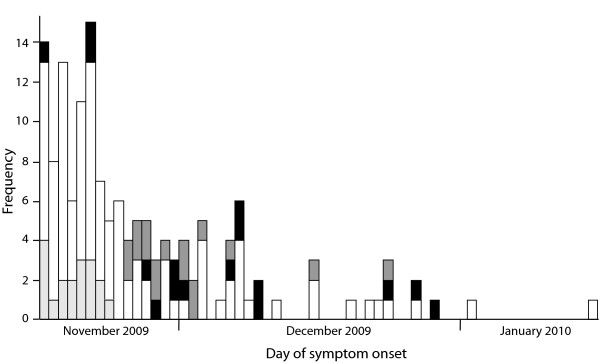
**Epidemiological curve of included cases by exposure**. Cases who were exposed (black), or assumed to be exposed (dark grey), and those whose vaccination was assumed to have taken place within 7 days before symptom onset (assumed unexposed; light grey) or who were unexposed (white) are presented by day of symptom onset. The assumption on exposure was based on cases with known date of vaccination. * For 1 patient, day of symptom onset was unknown. For this patient we used 1 day before day of admission as day of symptom onset for this epidemic curve.

A case and the matched controls were considered exposed (vaccinated) if they had received at least one pandemic influenza vaccination more than 7 days before symptom onset of the case [11,12,14,16]. The analysis was repeated for those who were vaccinated more than 14 days before symptom onset of the case. If the exact date of vaccination was unavailable, we extrapolated the time between vaccination date and symptom onset from vaccinated cases with a known date of vaccination. We assumed that the availability of the date of vaccination was independent of the day of symptom onset (see Figure 2). Therefore, we applied the percentage of unexposed cases among those with known vaccination date to vaccinees with unknown vaccination date. Furthermore, we assumed that vaccination less than 7 or 14 days before symptom onset (i.e. to be unexposed) was more likely in the beginning of November.

### Data analysis

Vaccine effectiveness was computed as VE = 1 - OR [27], with an exact 95% confidence interval (CI) around the point estimate. We used conditional logistic regression to calculate the odds ratio (OR). We used the VE estimate using the assumption on the validity of the vaccination based on the vaccinated cases with known date of vaccination (see exposure definition) as our primary analysis. Additionally, we used imputed delays between symptom onset and date of vaccination for those with unknown date of vaccination. We used multiple imputation (n = 10) and sampled from a uniform distribution with a lower bound of a delay of zero days and an upper bound of a delay of the sum of the number of days since the start of the study and 7 days. This upper bound is based on the start of the vaccination campaign relative to the start of this study. The overall estimates and 95%CI were determined using the method described by Rubin [28]. Furthermore, we considered all vaccinated cases with unknown vaccination date to be unexposed to yield the maximum VE.

Although we matched for underlying medical conditions, we were not able to match on the severity of these underlying medical conditions, as no such information was available for our cases. It could be argued that individuals hospitalised with pH1N1 suffer from more severe underlying medical conditions than community controls with underlying medical conditions. Therefore, we performed a *post hoc *sensitivity analysis in which controls were sampled from the pool of controls who received five or more different active drug compounds prescribed in the year prior (above median number) and matched on the same criteria as described before. The number of active drug compounds was therefore considered as a proxy of disease severity.

Data analysis was performed in SAS 9.2 (SAS Institute Inc. Cary, NC, USA).

## Results

### Sample characteristics

Of the 583 laboratory confirmed pH1N1 patients that were hospitalised during the study period, 459 (79%) had complete data on matching factors and history of vaccination. Only 149 were eligible for inclusion, as the majority of cases had no indication for pandemic influenza vaccination because of age (< 6 months; 19%) and/or absence of an underlying medical condition (74%).

The majority of the included cases fell ill in November 2009 (Figure 2), which coincided with the peak of the Dutch influenza epidemic. The median age of the included cases was 48 years (range 1-84), and 44% were male. Only 2% (n = 3) of the included cases was previously healthy (aged ≥ 60 years; of which two were assumed exposed); all others suffered from underlying medical conditions. Seven included cases died from pH1N1.

Forty-six percent (n = 68) of the cases reported to have obtained at least one pandemic influenza vaccination (Table 1). Among the vaccinated cases, exact date of vaccination was available for only 49% (n = 33). Vaccinees with or without available date of vaccination did not differ statistically on age (respectively 43 and 46 years old), sex (48% and 34% male) or the presence of underlying medical conditions (0/33 and 2/35). Furthermore, vaccinees without available date of vaccination who were assumed to be exposed did not differ from those who were assumed to be unexposed on age (respectively 51 and 57 years old), or the presence of underlying medical conditions (0/18 and 2/17). Respectively 48% and 33% of the vaccinated cases with exact date of vaccination available had obtained their vaccination more than 7 or 14 days before symptom onset (Table 1). Based on the extrapolation from available dates of vaccination, we assumed that 22% of the cases had received vaccination more than 7 days before the index date (exposed at symptom onset) and 15% more than 14 days before the index date. Of the seven fatal cases, four had obtained pandemic vaccination. For only one, date of vaccination was known. For this case, symptoms started five days after vaccination, too soon to expect an immune response from the vaccine. Two fatal cases (29%) were assumed to have been exposed. In controls, 46% had obtained at least one dose of pandemic influenza vaccination at the index date, of which 60% more than 7 days and 29% more than 14 days before the index date (Table 1). This resulted in a valid vaccination coverage of respectively 28% (7 days) and 13% (14 days) in controls. Because of the matching in which each stratum contains a single case, no direct comparison of vaccination coverage between cases and controls should be made.

**Table 1:** Vaccination history and completeness of data in cases, controls and controls with severe medical conditions.

	Used cut off for exposure	Cases	Controls	Controls with severe medical conditions
Crude vaccination coverage		46% (68/149)	46% (13012/28238)	53% (5752/10830)
Known vaccination date in vaccinees		49% (33/68)	100% (13012/13012)	100% (5752/5752)
Exposed cases (known vaccination date)	> 7 days > 14 days	48% (16/33) 33% (11/33)	60% (7798/13012) 29% (3715/13012)	58% (3320/5752) 27% (1551/5752)
Assumed exposed cases (unknown vaccination date)	> 7 days > 14 days	49% (17/35) 31% (11/35)	NA NA	NA NA
(Assumed) vaccination coverage	> 7 days > 14 days	22% (16+17/149) 15% (11+11/149)	28% (7798/28238) 13% (3715/28238)	31% (3320/10830) 14% (1551/10830)

Data using different cut offs for the validity of vaccination are presented. Note that no direct comparison of vaccination coverage between cases and controls should be made because of the matching in which each stratum contains a single case.

* NA = not applicable as for all controls, date of vaccination was available.

### Vaccine effectiveness

A considerable number of breakthrough infections was observed (Figure 2). The estimated VE was 19% (95%CI: -28-49; Table 2). The analysis with imputed data yielded a VE of 24% (95%CI -113-73). The maximum VE (all cases with unknown date of vaccination were assumed unexposed) was estimated at 74% (95%CI: 53-86).

**Table 2:** Effectiveness of the pandemic influenza vaccine in preventing 2009 influenza A/H1N1-related hospitalisation.

	VE (%) (95%CI)	
	**Exposed if > 7 days between vaccination and symptom onset**	**Exposed if > 14 days between vaccination and symptom onset**

VE*	19 (-28-49)	< 0 (upper 95%CI 30)
VE imputed data^$^	24 (-113-73)	26 (-160-79)
Restricted VE	49 (16-69)	35 (-26-66)
Restr. VE imputed^$^	51 (-43-83)	59 (-65-90)
Maximum VE^#^	74 (53-86)	61 (19-82)

The most realistic estimate of the vaccine effectiveness (VE), the VE estimation whereby we restricted our controls to those suffering from severe underlying medical conditions (restricted VE) and the maximum VE are presented. Results derived using different cut offs to define exposure are presented using the assumption based on cases with known date of vaccination and using imputed data.

* For 36 cases date of vaccination was missing. Respectively 49% and 31% of the vaccinated cases with unknown vaccination date were assumed to be exposed using 7 days or 14 days as cut off for the validity of vaccination (extrapolation from cases with known date of vaccination).

^$ ^We used multiple imputation and sampled (n = 10) from a uniform distribution with a lower bound of delay = 0 and an upper bound of delay = number of days since the start of the study + 7 days. This upper bound is based on the start of the vaccination campaign relative to the start of this study.

^# ^For the maximum VE, all vaccinees with unknown vaccination date were assumed unexposed.

The analysis using > 14 days as cut off for exposure revealed a VE of < 0% (upper 95%CI 30). In case data were imputed, the VE was estimated at 26% (95%CI -160-79; Table 2).

### Sensitivity analysis restricted to controls with severe underlying medical conditions

After restricting our analysis to controls who were prescribed at least five different types of medications (proxy for more severe underlying medical conditions), the control population included 10830 individuals (38% of original control sample; see Figure 1). The number of different prescriptions in the restricted population of controls ranged from 5 to 52, with a median of 7 prescriptions. Thirty-one percent of this selection of controls had obtained vaccination more than 7 days before the index date (Table 1), which resulted in a restricted VE of 49% (95%CI: 16-69; Table 2). Using imputed data, a similar result was obtained (VE = 51% (95%CI -43-83)). The maximum VE using the restricted control population was estimated at 84% (95%CI: 69-92).

## Discussion

This matched case-control study showed a considerable number of breakthrough infections, resulting in modest VE estimates. These results suggest that the MF-59™ adjuvanted vaccine may have had only a limited impact in preventing pH1N1-related hospitalisation in risk groups. Because pandemic vaccination started around the peak of the pH1N1 epidemic in the Netherlands, missing date of vaccination in hospitalised cases is a severe limitation to this study. Applying different scenarios partly overcame this limitation and provided a range of VE estimates, though, residual confounding by time cannot be excluded.

As pandemics occur unexpectedly, and during pandemics available resources could be heavily stretched, ideally routinely collected data should be used to provide estimates of VE against severe outcomes. We showed that using such data for VE estimates is feasible. However, observational studies to estimate the effectiveness of influenza vaccination are prone to bias [29-33]. Our study is not immune to such potential bias and although we matched for potential confounding variables, we had limited possibilities to adjust for residual confounding. The use of different types of routinely collected health care data and the consequent differences in the quality and level of information between cases and matched controls is a limitation of this study. In cases, vaccination status was self-reported whilst in controls vaccination was reported by the GP. GP registered data can be incomplete because of unreliable registration and because vaccination was also offered outside GP practices for certain individuals (e.g. those working in healthcare, those with children under the age of 6 months, children under the age of 5). By including controls who were eligible for vaccination by the GP and who were sampled from reliably-registering GPs we aimed to minimise potential underestimation of the vaccination coverage in controls. Recall bias in cases will have had only limited impact because of the short delay between the vaccination campaign and symptom onset, and because of the substantial attention of the general population for the pandemic influenza vaccine.

Frailty selection, resulting in confounding by severity, can be important in VE studies focussing on severe disease or hospitalisation as outcome [30,31]. Because the majority of the study population was relatively young (median age 48 years), and suffered from one kind of underlying medical condition, frailty is likely to have been of lesser importance relative to studies on seasonal influenza. Moreover, by matching our cases with controls on underlying medical conditions, we decreased the probability of confounding. However, because our cases could have been suffering from more severe underlying medical conditions than our matched controls, but without preventing them to obtain the vaccine, we performed a sensitivity analysis *post hoc *using controls with more severe underlying conditions. Using this restriction, the VE was estimated to be 35% (95%CI -26-66) or up to 59% (95%CI -65-90) depending on the cut off and method used to define exposure. However, it is known that several of our cases did not use any medication, suggesting only mild underlying medical conditions in those cases. These restricted VE estimates are therefore likely an overestimation of the actual VE.

The estimates of the effectiveness in preventing pH1N1-related hospitalisation of the adjuvanted pandemic influenza vaccines used in Europe ranged from 45% (95%CI 3-69; UK using a ASO3 adjuvanted vaccine [14]) to 90% (95%CI 48-100; Spain using several vaccines [18]) or even 100% (95%CI ∞-100; Scotland [13]). Even our maximum VE estimate was not as high as the Spanish and Scottish estimates. To determine the maximum VE, we assumed for all 35 cases with unknown date of vaccination (51% of all vaccinees) that vaccination took place within 7 days of symptom onset. This is unlikely a realistic assumption and therefore the maximum VE estimate is likely overestimated. A possible explanation for the wide range of VE estimates in Europe is the difference in inclusion criteria, the control group and the used vaccine. In Spain [18], the vaccine was mainly distributed to the usual influenza risk groups (including those with obesity), but all hospitalised patients were included in the study. The UK study [14] and our Dutch study focussed on the population most at risk for severe outcome of an influenza infection, and therefore only included individuals eligible for vaccination because of an underlying medical condition or advanced age. It is known that individuals with certain types of underlying medical condition or older age have a reduced response to vaccination [34,35]. A lower VE is therefore expected in this susceptible population. Furthermore, earlier published studies on VE against hospitalisation used the test-negative case-control design [14,18]. This design is susceptible to imperfect specificity and sensitivity of diagnoses and the vaccine coverage in test-negative hospital cases is possibly not representative for the general population. In addition, differential health care seeking behaviour between test-positives and test-negatives could result in biased estimates. As we used national data of notifications and data of an extensive GP network which are representative for the country, we expect that our cases and controls originate from the same, general population. However, our data have limited possibilities to refute the presence of potential bias which may have led to an underestimation of the VE.

The pandemic influenza vaccine used [19] contained half the amount of antigen relative to seasonal influenza vaccines plus an adjuvant to increase the immunogenicity. It is therefore not possible to make a direct comparison to seasonal influenza vaccines. However, the effectiveness of seasonal influenza vaccines in preventing influenza-related hospitalisations is also under debate. For seasonal influenza vaccination, a low VE estimate against hospitalisation (9-12%) was observed in those aged > 50 years using a difference-in-differences design [36]. Additionally, a Cochrane review concluded that seasonal influenza vaccination had no effect on hospital admissions or complication rates [17]. Taking into account the differences in study design and vaccines used, the effectiveness of influenza vaccines to prevent influenza-related hospitalisation appears lower than the effectiveness in preventing clinical disease [11-16]. The fact that those most at risk of complications and hospitalisations due to influenza react less favourably to the vaccine may have contributed to this difference. Adding the adjuvant to the vaccine did not overcome this problem in our population.

## Conclusion

In conclusion, the number of breakthrough infections resulting in a modest VE estimates suggests that the MF-59™ adjuvanted vaccine may have had only a limited impact on preventing pH1N1-related hospitalisation in this setting. As the main aim of influenza vaccination programmes is to reduce severe influenza-related morbidity and mortality from influenza in individuals at high risk of complications, a more effective vaccine, or additional preventive measures, are needed. Furthermore, efforts should be made to put better real-time monitoring systems in place to study the effectiveness of influenza vaccines in preventing severe laboratory-confirmed influenza.

## Competing interests

The authors declare that they have no competing interests.

## Authors' contributions

AS, EGW, JPD, MCS, MABvdS, and WvdH made substantial contributions to the concept and design, AS and EGW performed data acquisition, data analyses and interpretation of data, AS drafted the manuscript, EGW helped to draft the manuscript. All authors made important contributions to and read and approved the final manuscript.

## Pre-publication history

The pre-publication history for this paper can be accessed here:

http://www.biomedcentral.com/1471-2334/11/196/prepub
